# Origin of the crossover from polarons to Fermi liquids in transition metal oxides

**DOI:** 10.1038/ncomms15769

**Published:** 2017-06-08

**Authors:** Carla Verdi, Fabio Caruso, Feliciano Giustino

**Affiliations:** 1 Department of Materials, University of Oxford, Parks Road, Oxford OX1 3PH, UK

## Abstract

Transition metal oxides host a wealth of exotic phenomena ranging from charge, orbital and magnetic order to nontrivial topological phases and superconductivity. In order to translate these unique materials properties into device functionalities these materials must be doped; however, the nature of carriers and their conduction mechanism at the atomic scale remain unclear. Recent angle-resolved photoelectron spectroscopy investigations provided insight into these questions, revealing that the carriers of prototypical metal oxides undergo a transition from a polaronic liquid to a Fermi liquid regime with increasing doping. Here, by performing *ab initio* many-body calculations of angle-resolved photoemission spectra of titanium dioxide, we show that this transition originates from non-adiabatic polar electron–phonon coupling, and occurs when the frequency of plasma oscillations exceeds that of longitudinal-optical phonons. This finding suggests that a universal mechanism may underlie polaron formation in transition metal oxides, and provides a pathway for engineering emergent properties in quantum matter.

## Abstract (web summary)

Photoemission spectroscopy studies of anatase titanium oxide have demonstrated a doping-driven crossover in the polaronic character of charge carriers. Here, the authors obtain a first principles understanding of these observations in terms of plasma screening and electron–phonon coupling.

Elucidating the nature of charge carriers in doped transition metal oxides (TMOs) is key to understanding the mechanism of electrical conduction in these multifunctional materials. In conducting oxides the infrared-active vibrations can couple strongly to electrons, leading to the formation of polarons^1^. Polarons are electrons dressed by a phonon cloud^2^, and represent a paradigmatic example of emergent state in condensed matter. Depending on their mass and size, polarons exhibit widely different conduction mechanisms, from band-like transport to thermally activated hopping transport^3^,^4^. Despite being central to the science and technology of oxides, little is known about the properties of polaronic states.

The interest in electron–phonon coupling and polaronic quasiparticles in TMOs has been reinvigorated by recent angle-resolved photoelectron spectroscopy (ARPES) experiments^5^,^6^,^7^,^8^,^9^. The signature of polaronic behaviour in ARPES spectra is the appearance of satellites below the conduction band, at integer multiples of the optical phonon energy. This is reported in Fig. 1a,b for the paradigmatic case of doped anatase TiO_2_ (ref. 5). These pioneering measurements showed that by increasing the carrier concentration, polaronic satellites gradually evolve into the photoemission kinks observed in metals and superconductors^10^ (see Fig. 1c). It was proposed that this crossover reflects the evolution of charge carriers from polarons to a Fermi liquid^5^,^8^. In order to clarify the origin of this transition without making any *a priori* assumption about the underlying mechanism, first principles calculations are urgently called for. However, the investigation of polaronic features in ARPES spectra from first principles and their evolution with doping is exceptionally challenging and has never been reported before.

In the following we focus on the prototypical example of anatase TiO_2_. On top of its well-known applications in solar energy harvesting^11^,^12^ and superhydrophilic technology^13^,^14^, this material is also being investigated in the quest for transparent conducting oxides based on non-toxic and Earth-abundant elements^15^,^16^. Despite its pivotal role in a broad range of technologies, the nature of the charge carriers in anatase is still controversial^17^. Here we address these issues by calculating ARPES spectra and polaron wavefunctions entirely from first principles. We develop a theoretical and computational framework that allows us to investigate polarons and Fermi liquid quasiparticles on the same footing, and without resorting to any empirical parameters. Using this approach, we show how the interplay between the dynamical screening of the electron plasma and the Fröhlich electron–phonon coupling is responsible for the transition between polaronic and Fermi liquid states. We propose that the mechanism identified in this work may be universal, and also applies to other oxides such as SrTiO_3_ and ZnO.

## Results

### Angle-resolved photoemission spectra

**Figure 1:**
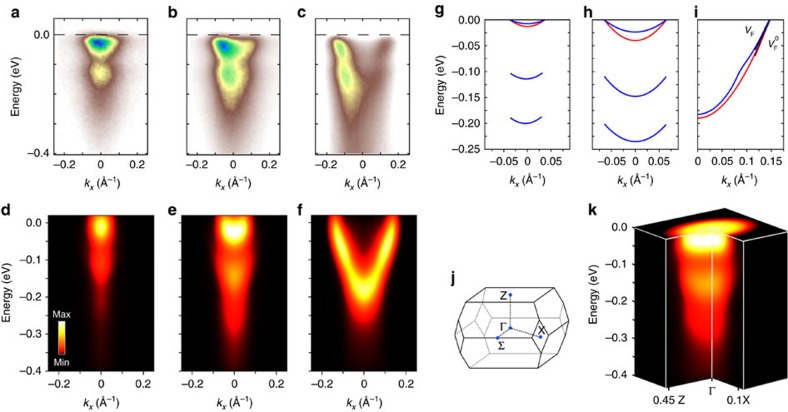
*Ab initio* ARPES spectra of *n*-doped anatase TiO_2_. (**a**–**c**) ARPES spectra of anatase TiO_2_ measured by Moser *et al*.^5^. The measurements were taken at 20 K on samples with 5 × 10^18^ cm^−3^ (**a**), 3 × 10^19^ cm^−3^ (**b**) and 3.5 × 10^20^ cm^−3^ (**c**). The zero of the energy is set to the Fermi level. The electron momentum *k*_*x*_ is along the ΓΣ line of the anatase Brillouin zone (see **j**). Reproduced with permission from ref. 5. Copyright 2013 by the American Physical Society. (**d**–**f**) Calculated spectral function of anatase TiO_2_, for the same electron momenta and nominal doping levels as in **a**–**c**. Gaussian masks of widths 25 meV and 0.015 Å^−1^ were applied to account for the experimental resolution^5^. (**g**–**i**) Band structures extracted from the calculated spectral functions in **d**–**f**. The bare bands are in red, the bands including electron–phonon interactions are in blue. The calculated mass enhancement parameter *λ* is 0.73 (**g**), 0.70 (**h**) and 0.20 (**i**). (**j**) Brillouin zone and high-symmetry lines of anatase TiO_2_. (**k**) Calculated ARPES spectrum for a doping concentration of 3 × 10^19^ cm^−3^, showing the anisotropy of the electron dispersions along ΓX (basal plane of the tetragonal lattice, see Supplementary Fig. 1) and ΓZ (*c*-axis).

Our calculated ARPES spectra are shown in Fig. 1d–f, for the same doping levels as in the measurements of ref. 5, reproduced in Fig. 1a–c. These maps show the bottom of the conduction band of *n*-doped anatase TiO_2_, for three doping levels in the range 10^18^–10^20^ cm^−3^. All the spectra exhibit a bright parabolic band, whose size increases with doping. This reflects the rise of the Fermi energy inside the conduction band as the electron density increases. Besides this bright feature, panels a–b (experiments) and d–e (calculations) show each a pair of satellites, a bright one at a binding energy around 0.1 eV, and a dim one near 0.25 eV. These features are identified as polaronic effects^5^. Moving on to higher doping in panels c and f, the satellites disappear and are replaced by band structure kinks near 0.1 eV. Overall, our calculated ARPES spectra are in remarkable agreement with the experiments of ref. 5. In order to achieve this unprecedented level of precision without any adjustable parameters, we developed an innovative computational framework.

In our calculations the photoelectron intensity maps are obtained using the single-particle spectral function *A*(**k**, *ω*), where *ħ***k** and *ħω* are the crystal momentum and binding energy, respectively, and *ħ* is the reduced Planck constant. The spectral function is calculated using the state-of-the-art cumulant expansion, which has been employed recently to successfully describe plasmon satellites^18^,^19^,^20^,^21^. The cumulant expansion can naturally be applied to investigate the spectral properties of a polaronic system since the theory stems from the exact solution of the Fröhlich electron–boson coupling Hamiltonian^22^,^23^. In this formalism the spectral function is expressed as ref. 24:


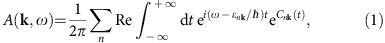


where *n* is the electron band index, *ɛ*_*n***k**_ is the electron eigenvalue in absence of many-body interactions, and *t* is the time variable. The quantity *C*_*n***k**_ is the so-called cumulant function, and can be calculated by using the standard electron–phonon self-energy *Σ*_*n***k**_ as a seed (see Methods and Supplementary Note 1):


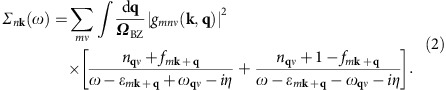


Here *g*_*mnν*_(**k**, **q**) is the electron–phonon vertex, and describes the probability amplitude for an electron in the initial state 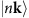 to be scattered into the final state 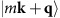 by a phonon with momentum *ħ***q** and energy *ħω*_**q***ν*_ in the branch *ν* (ref. 25). The terms *n*_**q***ν*_ and *f*_*m***k**+**q**_ denote the Bose–Einstein and Fermi–Dirac occupations, respectively; *Ω*_B*Z*_ is the Brillouin zone volume, and *η* a positive infinitesimal. The numerical evaluation of equations (1) and (2) is very challenging owing to the singular nature of the Fröhlich interaction at long wavelengths^26^. To overcome this challenge we use the Wannier function technique of refs 27, 28, as implemented in the EPW code^29^.

The strength of the electron–phonon interaction is most commonly expressed in terms of a single parameter *λ*, which describes the enhancement of the electron mass from the band effective mass *m*_b_ to the polaron mass m* via m*=*m*_b_(1+*λ*), as well as the renormalization of the Fermi velocity^30^. To determine this parameter we first extract the band structures underlying Fig. 1d–f, by tracking the maxima in the energy distribution curves. This procedure yields a set of parabolic bands and their satellites in Fig. 1g,h, and a distorted parabola in Fig. 1i (blue curves). For comparison we also report the electronic bands in absence of electron–phonon interactions (red curves). For carrier concentrations of 5 × 10^18^ and 3 × 10^19^ cm^−3^, polaron satellites are clearly visible in Fig. 1g,h, whereas at 3.5 × 10^20^ cm^−3^ we see a band structure kink but no satellites. From these band structures we obtain the mass enhancement parameter as the ratio between the Fermi velocity of the bare band, 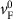, and that of the dressed band, *v*_F_, as indicated in Fig. 1i: 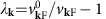 (ref. 30), where we explicitly included the dependence on the wavevector at the Fermi surface. As we move from the lowest to the highest doping level we obtain *λ*_**k**_=0.73, 0.70 and 0.20, respectively. Our calculated value at intermediate doping is in excellent agreement with the mass enhancement determined in experiments, *λ*=0.7 (ref. 5).

In Fig. 1k we show the spectrum calculated at intermediate doping for electron momenta along ΓX and ΓZ, as well as the Fermi surface cut. Here we see that the Fermi surface pocket is elongated along the ΓZ direction. This elongation reflects the anisotropic character of the band effective masses, which we calculate to be 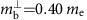 and 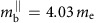 along ΓX and ΓZ, respectively (*m*_e_ is the electron mass). Surprisingly this anisotropy is not reflected in the electron–phonon coupling strength: our calculations indicate that the mass enhancement *λ*_*k*_ varies by less than 10% along the [100], [110] and [001] directions, leading to an average value of *λ*=0.73, 0.70 and 0.19 for the three doping levels considered (see also Supplementary Note 2). These results are in good agreement with resonant inelastic X-ray scattering experiments on anatase TiO_2_ (ref. 31).

### Origin of satellites and kinks

**Figure 2:**
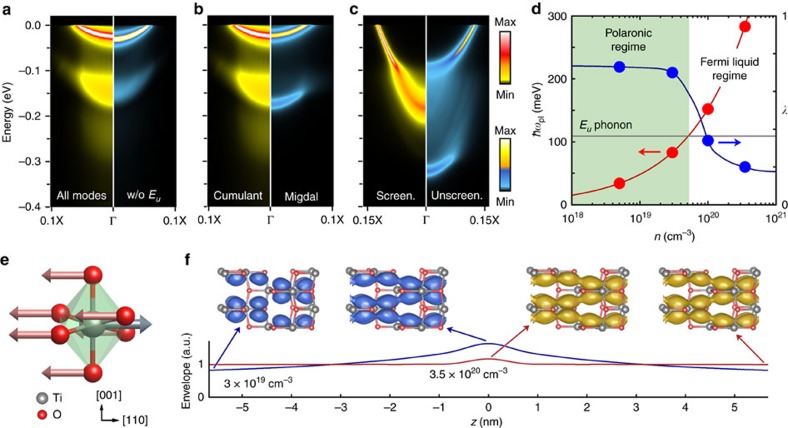
Origin of satellites and kinks, and polaron wavefunctions. (**a**) Effect of high-energy phonons: we compare the spectral function calculated for the intermediate doping level (3 × 10^19^ cm^−3^) by taking into account all vibrational modes and a calculation where all phonons with energy above 100 meV have been eliminated. (**b**) Effect of electron–phonon correlations beyond the one-shot Migdal approximation: the complete calculation using the cumulant expansion method is compared to a spectral function calculated within the one-shot Migdal approximation. The doping level is the same as in **a**. (**c**) Effect of dynamical screening on the spectral function: we compare the spectral function calculated for the highest doping level (3.5 × 10^20^ cm^−3^) by taking into account the screening of the electron–phonon interaction by carriers, with a calculation where this effect is turned off, and as a result the electron–phonon coupling is artificially enhanced. For clarity these spectra were not convoluted with Gaussian masks as in Fig. 1d–f. (**d**) Identification of polaronic region and Fermi liquid region in *n*-doped anatase TiO_2_: the red spheres represent the plasmon energy at each doping level, the horizontal line is the energy of the LO *E*_*u*_ phonon (109 meV). The electron–phonon coupling strength *λ* is given by the blue spheres. The lines are guides to the eye. (**e**) Ball-and-stick representation of the LO *E*_*u*_ phonon, showing for clarity only one of the TiO_6_ octahedra. (**f**) Square moduli of the polaron wavefunctions near the origin and further away from the origin, in the polaronic region (blue, 3 × 10^19^ cm^−3^) and in the Fermi liquid region (yellow, 3.5 × 10^20^ cm^−3^). The corresponding envelope functions are shown as the blue and red curves, respectively. These wavefunctions are extended over all three Cartesian directions, but are only shown along the *c*-axis for clarity.

Having established that our calculations can accurately reproduce experimental spectra without adjustable parameters, we now proceed to identify the mechanisms that drive the formation of polaron satellites by selectively turning off individual components of the calculations. The energy separations between the quasiparticle bands and the first satellite in Fig. 1d,e are 106 and 124 meV, respectively. In the case of Fig. 1f the kink appears at a binding energy of 100 meV. These energy scales are compatible with a Fröhlich-type coupling to the longitudinal-optical (LO) *E*_*u*_ phonon at 109 meV (see phonon dispersions in Supplementary Fig. 1). This vibrational mode corresponds to the stretching of the Ti–O bonds in the *ab* plane, as shown in Fig. 2e. Another candidate bosonic mode is the *c*-axis *A*_2*u*_ phonon at 88 meV (ref. 31); however, the energy of this mode appears too small to account for the satellites and kinks in Fig. 1. In order to quantify the importance of the *E*_*u*_ phonon, in Fig. 2a we compare two calculations: the complete spectrum at intermediate doping and a calculation where the coupling to modes with energy above 100 meV is artificially suppressed. We see that, upon removing high-energy phonons, the intensity of the first satellite decreases and the second satellite disappears. The effective mass is also visibly lower; in fact, the analysis of the mass enhancement parameter yields *λ*=0.3, to be compared to the total coupling *λ*=0.7. It follows that the *E*_*u*_ phonon contributes ∼60% of the total coupling, hence it represents the primary mechanism behind the satellites.

In Fig. 2b we test the importance of many-body correlations beyond the one-shot Migdal approximation. The spectrum at intermediate doping, obtained from the cumulant expansion, is shown together with the result of a calculation within the one-shot Migdal approximation (see Methods). The Migdal approximation is obtained from the complete electron–phonon self-energy by neglecting the three-point vertex^2^. In this approximation the electron–phonon self-energy contains only non-crossing diagrams. The additional approximation adopted in first principles calculations is to replace the fully renormalized electron and phonon propagators by those evaluated within density functional theory (DFT)^25^. We name this choice one-shot Migdal approximation to emphasize the lack of self-consistency in the electron propagator. From Fig. 2b we notice that the one-shot Migdal approximation fails twice: firstly, the separation between the quasiparticle band and the first satellite is too large as compared to experiment (151 meV instead of ∼100 meV); secondly, the dim satellite around 0.25 eV is completely missing. This test highlights the crucial role of high-order electron–phonon correlations in the description of polarons in conducting oxides. It would be interesting to see whether the deficiencies of the one-shot Migdal approximation could be avoided by performing a fully self-consistent Migdal calculation^32^, and to establish the importance of crossing diagrams which are included in the cumulant expansion; however this test is currently out of reach.

We now move to discuss the origin of the crossover between satellites and kinks in the spectra as a function of doping. The Fröhlich theory of polarons^26^ considers a single electron added to a polar insulator. This description does not take into account that, as more electrons are added to the system, the polar electron–phonon coupling is weakened by the electronic screening of the charge carriers. Following ref. 2, we treat this issue by screening the electron–phonon vertex by the frequency-dependent Lindhard dielectric function with the calculated effective mass and dielectric permittivity of anatase TiO_2_ (see also Methods). For completeness we show in Supplementary Fig. 2 the dielectric screening as a function of carrier density and how the Fröhlich electron–phonon vertex is influenced by doping. In order to illustrate the importance of carrier screening, in Fig. 2c we compare two scenarios: the spectrum calculated at high doping by accounting for electronic screening and the same system, but this time ignoring the screening of the electron–phonon coupling by doped carriers. The result is striking: in the absence of screening one obtains a sharp polaron satellite, in stark disagreement with experimental evidence. This comparison indicates that a correct description of the electronic screening is absolutely crucial to capture the photoemission kink at high doping. On the contrary, when we repeat the comparison of Fig. 2c for the cases of low and intermediate doping, we find a completely different picture: at these doping levels the electronic screening does not play any significant role.

These conflicting observations can be rationalized by inspecting the timescales of lattice vibrations and electronic screening. The *E*_*u*_ phonon vibrates with a period *T*_ph_=38 fs. The characteristic response time of the electronic screening is set by the plasma frequency of doped carriers, *T*_el_=2*π*/*ω*_p_. In the case of *n*-doped TiO_2_ the electrons occupy a singly degenerate parabolic band minimum; therefore 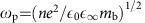, where *n* is the electron density, 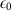 the permittivity of vacuum and 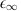 the high-frequency dielectric constant of TiO_2_ (ref. 2). For the electron densities considered in Fig. 1a–c we calculate *T*_e*l*_=122, 50 and 15 fs, respectively. From these values we deduce that, at the lowest doping, the carriers are too slow to screen the long-range electric field generated by the oscillation of the *E*_*u*_ phonon (122 fs versus 38 fs). In this case the screening is ineffective and the Fröhlich interaction dominates the spectrum. On the contrary, at the highest doping the electrons oscillate faster than the LO phonon (15 fs versus 38 fs). In this case the electronic screening is almost complete and the Fröhlich coupling is largely suppressed. In this regime the strength of the kink depends critically on the carrier concentration; with increasing doping the coupling to the LO phonons is gradually suppressed, and the ARPES spectrum is dominated by the weaker coupling of carriers to non-polar phonons^8^. These considerations are summarized in Fig. 2d, where we compare the energy of the *E*_*u*_ phonon with the plasma energy *ħω*_p_ of the carriers, and we monitor the evolution of the coupling strength with doping. We can identify two regions: a polaronic regime, corresponding to the situation *ω*_p_<*ω*_ph_, and a Fermi liquid regime, corresponding to *ω*_p_>*ω*_ph_. In the polaronic region the electronic screening is ineffective, we see satellites in the spectra, and the electron–phonon mass enhancement is not sensitive to the doping level. In the Fermi liquid region the Fröhlich coupling is strongly suppressed, polaron satellites are gradually replaced by photoemission kinks, and the coupling strength decreases. To further validate this trend, we performed additional calculations for a doping concentration of 1 × 10^20^ cm^−3^ in the transition region. We found that one satellite is still present in the spectrum (Supplementary Fig. 3). A careful investigation of the mass renormalization parameter yields *λ*=0.34, thus confirming that the electron–phonon coupling is weakened by the electronic screening. The present analysis reveals that the origin of the crossover from polarons to a Fermi liquid in the ARPES spectra of doped TiO_2_ is to be found in a form of electron–phonon coupling, which we refer to as a non-adiabatic Fröhlich interaction.

Given the qualitative change in the band structures at the crossover from the polaronic to the Fermi liquid regime, it is natural to ask how this evolution is reflected in the wavefunctions. In order to explore this aspect we calculate the polaron wavefunctions by generalizing the perturbation theory of ref. 2 to *ab initio* calculations. Figure 2f shows the square moduli of the polaron wavefunctions at the bottom of the conduction band near the maximum of the polaron wavefunction and a few unit cells away: in blue the intermediate doping level, which is inside the polaronic region of Fig. 2d; in yellow the high doping level, in the Fermi liquid region. The corresponding envelope functions are also shown. Here we see that the wavefunction of an electron in the Fermi liquid region is essentially akin to a periodic Bloch function. On the contrary, the wavefunction of an electron in the polaronic region shows spatial localization. To quantify the size of the polaron in this latter case we define a polaron radius *r*_p_ using the half width at half maximum. From Fig. 2f we obtain *r*_p_=5.7 nm. This result indicates that, despite the qualitative changes in the ARPES spectra, we are in the presence of large polarons throughout the entire doping range, and that polarons in TiO_2_ are considerably more delocalized than previously thought^5^. Since thermally activated hopping transport corresponds to *r*_p_ of the order of the lattice constant^33^, our finding supports the notion that electrical conduction in anatase TiO_2_ takes place via standard band-like transport. To avoid ambiguities we emphasize that the present result refers to intrinsic mobile polarons, not to localized electronic defect states such as those associated with O vacancies and which are not mobile^34^.

## Discussion

The non-adiabatic Fröhlich mechanism identified here is simple enough that it is likely to play a role in many other conducting oxides. In order to test this hypothesis we estimate the critical density for the crossover in doped SrTiO_3_ (ref. 8) and doped ZnO (ref. 9). In SrTiO_3_ the plasma energy for an electron concentration of 10^20^ cm^−3^ is *ħω*_p_∼64 meV (ref. 35); therefore, to match the 100 meV LO phonon of SrTiO_3_ one would need a doping level of 2.6 × 10^20^ cm^−3^. In the experiments of ref. 8 the carriers are confined in a thin surface layer corresponding to approximately three unit cells; therefore, we estimate the critical carrier density for the two-dimensional electron liquid in the range 3 × 10^13^ cm^−2^. This value is remarkably close to the critical density determined experimentally, 4 × 10^13^ cm^−2^ (ref. 8). More accurate calculations will need to take into account the two-dimensional screening at the surface of SrTiO_3_ and its quantum paraelectric nature. A similar analysis can be carried out for ZnO (ref. 9). In this case the energy of the highest LO phonon is 72 meV, while the plasma energy for a surface electron concentration of 7.5 × 10^13^ cm^−2^ is 320 meV (ref. 36). The transition would then appear around 3.8 × 10^12^ cm^−2^, slightly below the density reported in ref. 9. In agreement with our estimate, the spectra of ref. 9 exhibit a behaviour which is intermediate between kinks and satellites.

In sum, our findings indicate that the electron–phonon coupling in TiO_2_ is more complex than previously thought, and that the non-adiabatic Fröhlich coupling could be the unifying mechanism behind the transition from polarons to Fermi liquids in conducting oxides; explicit *ab initio* calculations will be required to confirm this point. Looking further ahead, our work suggests that a fine control of the interplay between lattice vibrations and plasma oscillations may offer a pathway for investigating emergent states in quantum materials, and provide opportunities in the development of quantum technologies based on TMOs.

## Methods

### Ground-state calculations

*Ab initio* calculations were carried out for anatase TiO_2_ (space group *I*4_1_/*amd*), using the experimental lattice parameter *a*=3.784 Å (ref. 37). We used DFT within the generalized gradient approximation of Perdew, Burke and Ernzerhof^38^. The core–valence interaction was described by means of norm-conserving pseudopotentials, with the semicore Ti-3*s* and Ti-3*p* states explicitly taken into account. Electron wavefunctions were expanded in a planewaves basis set with kinetic energy cutoff of 200 Ry, and the Brillouin zone was sampled using a 6 × 6 × 6 Monkhorst–Pack mesh. Lattice-dynamical properties were calculated using density functional perturbation theory (DFPT). All DFT and density functional perturbation theory calculations were performed using the Quantum ESPRESSO^39^ package.

### Electron–phonon coupling

Calculations of electron–phonon couplings were performed using the EPW code^29^, the cumulant expansion was performed separately (see Supplementary Note 1). The Fröhlich electron–phonon matrix element was calculated using the method of ref. 28. An accurate description of the Fröhlich vertex as described in ref. 28 is essential: ignoring this effect leads to a severe underestimation of the mass enhancement^40^. To evaluate equation (2) we computed electronic and vibrational states as well as the scattering matrix elements on a 4 × 4 × 4 Brillouin-zone grid. These quantities were interpolated with *ab initio* accuracy onto a fine grid with 2·10^6^ random *q*-points using EPW. The positive infinitesimal *η* was set to 10 meV. Temperature effects were accounted for by including the Fermi–Dirac occupation in the spectral function, corresponding to the experimental temperature of 20 K.

### Doping

Doping was included using the rigid-band approximation since the system is degenerate. In fact, by considering the Mott criterion for the metal-insulator transition^41^, 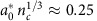 where 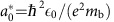 is the effective Bohr radius, we obtain a value *n*_*c*_=1.3 × 10^18^ cm^−3^ for the critical density that is below the doping levels investigated. The screening of the electron–phonon interaction arising from the doped carriers was taken into account by computing the dielectric function 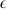
(**q**, *ω*) in the random-phase approximation^2^,^42^, for a homogeneous electron gas with the calculated effective mass *m*_*b*_ and dielectric permittivity 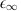 of anatase TiO_2_ (refs 2, 42) (see Supplementary Note 3 for a rationale). In this expression the plasma frequency directly reflects the carrier density; therefore, the influence of doping on the dielectric screening in anatase TiO_2_ (ref. 43) is included in the calculations. The resulting non-adiabatic matrix element is 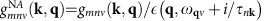, where *τ*_*n***k**_ is the electron lifetime near the band edge. Here we use *ħ*/*τ*_*n***k**_=55 meV from ref. 5. We checked that this choice does not affect our conclusions (Supplementary Fig. 4). We note that, owing to the strong anisotropy of the Fermi surface in *n*-doped anatase TiO_2_, it is possible that the nominal doping levels estimated in ref. 5 using the Fermi momentum may underestimate the actual carrier densities in the measured samples.

### Angle-resolved photoemission spectra

The cumulant function used in the calculation of the ARPES spectra is obtained from the self-energy of equation (2) as follows^18^: 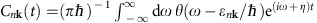 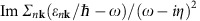, where *θ* is the Heaviside function. More details on the cumulant expansion are provided in Supplementary Note 1. The calculations within the one-shot Migdal approximation shown in Fig. 2b were performed by using directly equation (2) inside the spectral function^25^: 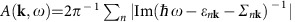. The spectra were computed without including photon matrix elements effects^10^. This approximation is justified since the variation of the orbital character over the ∼0.1 eV binding energy range shown in Fig. 1 is negligible^10^. In order to extract the bands shown in Fig. 1g–i we proceeded as follows. For the quasiparticle bands, which are the brightest features in Fig. 1d–f, we calculate the poles of the electron Green's function as *E*_*n***k**_=*ɛ*_*n***k**_+Re *Σ*_*n***k**_(), where *ɛ*_*n***k**_ and *E*_*n***k**_ are the bare and the renormalized dispersions, respectively. For the satellites in Fig. 1d,e the previous procedure is not applicable since these features are not poles of the Green's function. In this case we directly identify the maxima in the energy dispersion curves^10^, and obtain the pairs of parabolic bands which are visible in Fig. 1g,h at binding energies below 0.1 eV.

### Polaron wavefunction

In order to calculate the wavefunction of the polaron we generalized the perturbation theory approach of ref. 2 to the case of multiple electron bands and phonon branches, as well as *ab initio* electronphonon matrix elements. We found 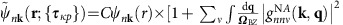 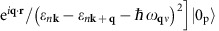, where *ψ*_*n***k**_ and 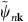 are the electron wavefunction without and with electron–phonon interactions, respectively. 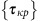 indicate the nuclear coordinates, 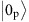 is the phonon ground state and *C* is a normalization constant. The details of the derivation are given in Supplementary Note 4. The wavefunctions in Fig. 2f were obtained by setting *n***k** to the conduction band edge; the envelope functions were calculated by retaining only the term inside the square brackets in the above expression. A random grid with 2 × 10^6^ points was used for the Brillouin-zone integration, and a small imaginary part *η*=10 meV was added to the denominator.

### Data availability

The data that support the findings of this study are available from the corresponding author on request, as is the modified version of the EPW package to include screening and the custom code for the cumulant expansion.

## Additional information

**How to cite this article:** Verdi, C. *et al*. Origin of the crossover from polarons to Fermi liquids in transition metal oxides. *Nat. Commun.* **8,** 15769 doi: 10.1038/ncomms15769 (2017).

**Publisher's note**: Springer Nature remains neutral with regard to jurisdictional claims in published maps and institutional affiliations.

## Supplementary Material

Supplementary Information Supplementary Figures, Supplementary Notes and Supplementary References
