# Supplementary material for: Origin of the crossover from polarons to Fermi liquids in transition metal oxides
Source: Nat Commun. 2017 Jun 8;8:15769. doi: 10.1038/ncomms15769 (PMC5472750; doi:10.1038/ncomms15769)
Supplement: Supplementary Information — Supplementary Figures, Supplementary Notes and Supplementary References [file ncomms15769-s1.pdf]

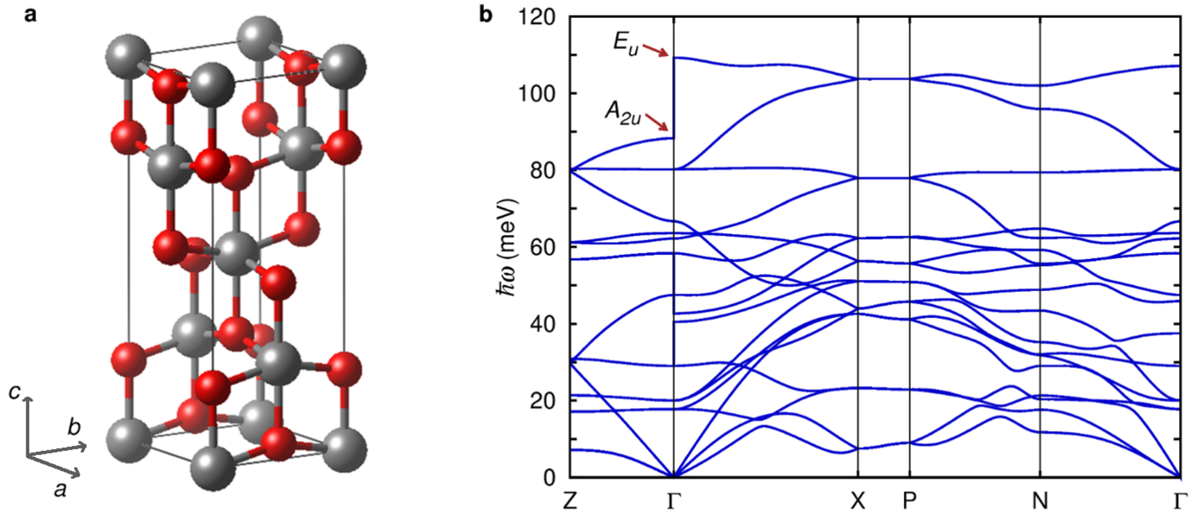

Supplementary Figure 1 | Crystal structure and phonon dispersions of anatase TiO_2. (**a**) Ball-and-stick model of the tetragonal unit cell of anatase TiO_2. Ti atoms are in gray and O atoms are in red. The lattice vectors and crystallographic directions referred to in the main text are indicated. (**b**) Calculated phonon dispersion relations of anatase TiO_2 along high-symmetry directions of the Brillouin zone. The E_u and A_{2u} phonons discussed in the main text are indicated. The A_{2u} phonon is infrared active only along the c axis, accordingly it is found to give a smaller contribution to the total Fröhlich coupling after integrating over all phonon momenta.

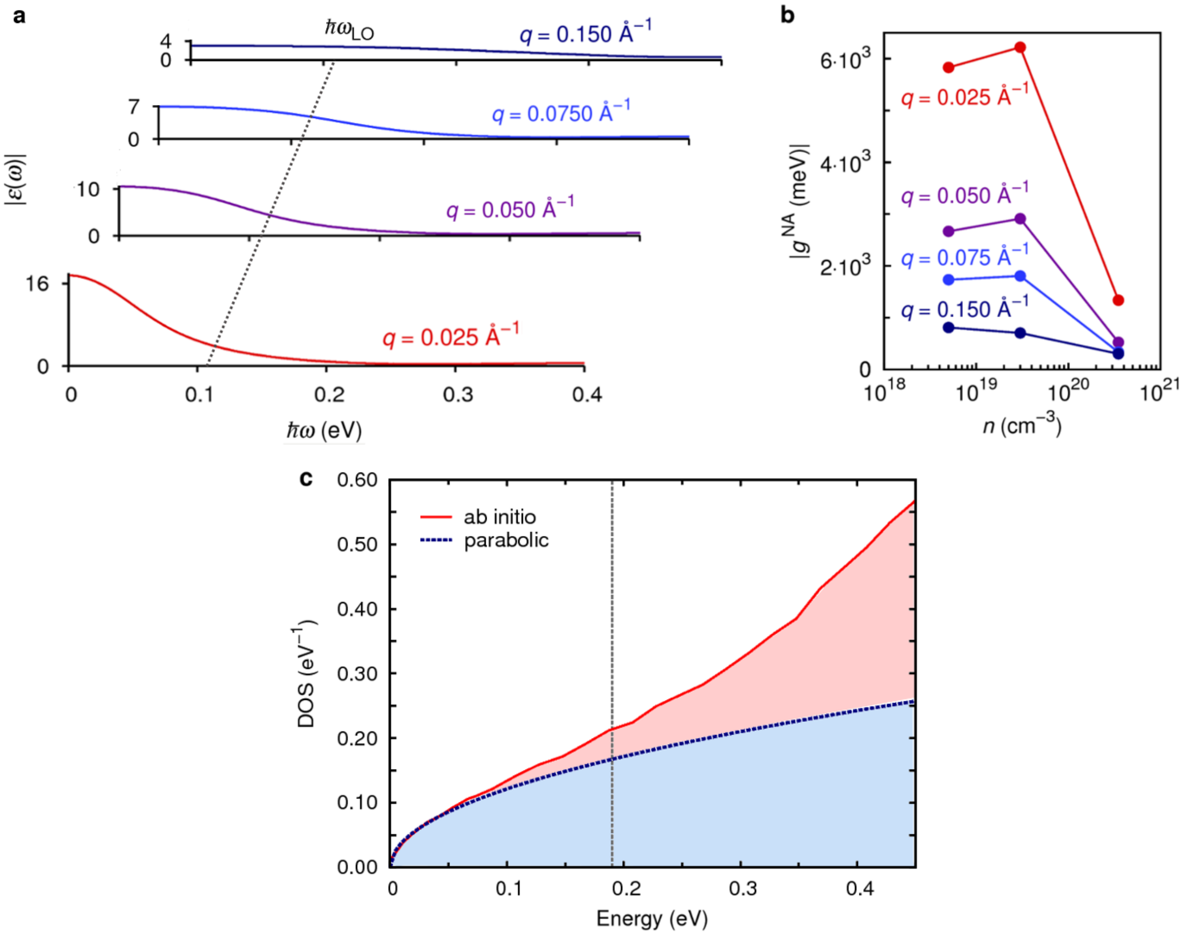

Supplementary Figure 2 | Non-adiabatic electron-phonon matrix elements in anatase TiO_2. (**a**) Lindhard dielectric function associated with doped carriers in anatase TiO_2 at different values of $\mathbf{q}$. For illustration we consider the highest doping level, 3.5×10^{20} cm^{-3}. (**b**) Non-adiabatic electron-phonon matrix elements $g^{\mathrm{NA}}_{mn\nu}(\mathbf{k},\mathbf{q})$ corresponding to the LO E_u phonon of anatase TiO_2, as a function of doping level. We set $n\mathbf{k}$ to the bottom of the conduction band. The electron-phonon matrix element becomes weaker at high doping. (**c**) Comparison between the density of states (DOS) near the conduction band bottom calculated from first principles (red) and modelled with a parabolic band (blue): $\mathrm{DOS}(E) = V/(2\pi^2)\,(2m_{\mathrm{b}}/\hbar^2)^{3/2}\sqrt{E}$, where V is the unit cell volume of anatase. The *ab initio* DOS was computed using Wannier interpolation to obtain the electronic energies on a randomly generated dense $\mathbf{k}$ grid and with a Gaussian smearing of 5 meV. The gray vertical line indicates the energy corresponding to the Fermi level at the highest doping. The parabolic DOS starts deviating significantly from the calculated one for energies above 0.2 eV.

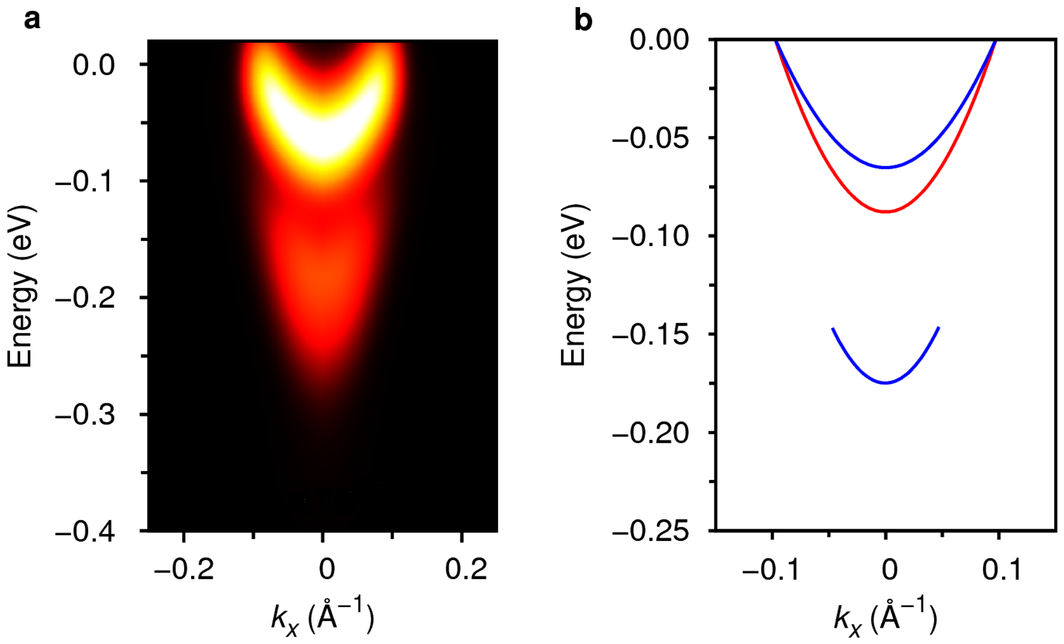

Supplementary Figure 3 | Spectral function in the transition regime. (**a**) Spectral function of anatase TiO_2 calculated for the doping level 1×10^{20} cm^{-3}. Gaussian masks of widths 25 meV and 0.015 Å^{-1} were applied as in Fig. 1 of the main text. (**b**) Band structure extracted from **a** (blue lines) together with the bare band (red line). For this doping one satellite is still visible but the mass renormalization parameter is decreasing ($\lambda = 0.34$ as reported in the main text).

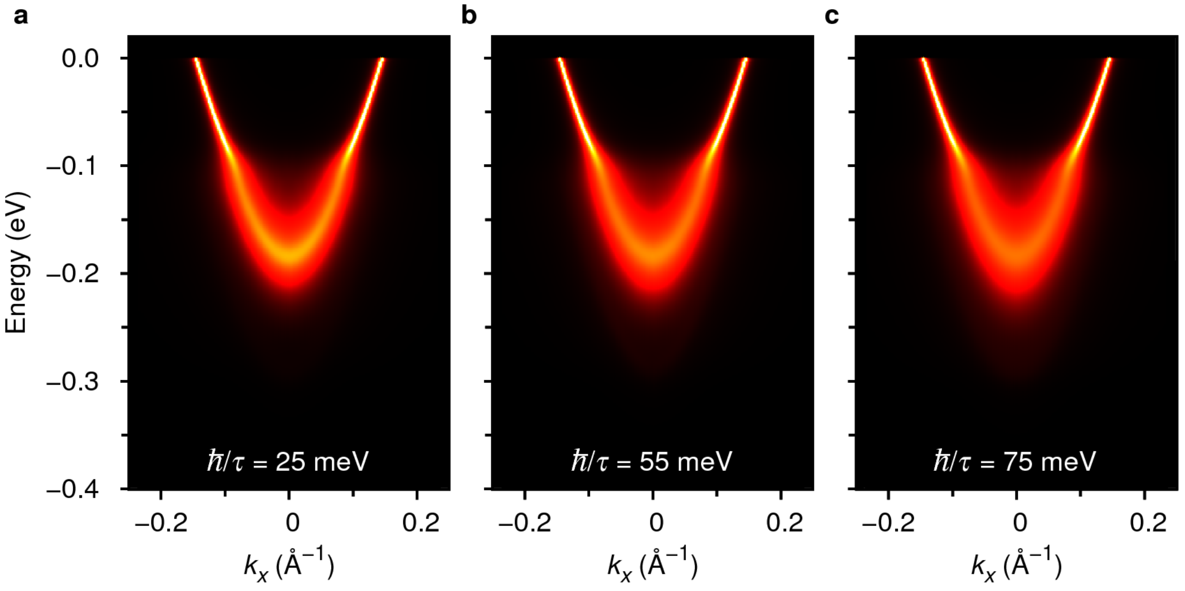

Supplementary Figure 4 | Impact of the electron lifetime on the spectral properties. Spectral function of anatase TiO_2 calculated for the doping level 3.5×10^{20} cm^{-3} using $\hbar/\tau = 25$ (**a**), 55 (**b**) and 75 meV (**c**) to compute the electronic screening (see Methods). The spectral features are virtually unchanged. We recomputed the mass renormalization parameter λ as in the main text which gives $\lambda = 0.19$, 0.20 and 0.22 for increasing broadening. The same analysis for the doping concentration 5×10^{18} cm^{-3} yields $\lambda = 0.77$, 0.73 and 0.72 for the same broadenings. These values fall within 10% of the results presented in the main text.

Supplementary Note 1 | Cumulant expansion

The spectral function is related to the imaginary part of the one-electron retarded Green's function by:

$$A(\mathbf{k},\omega) = -\frac{1}{\pi}\sum\nolimits_n \mathrm{Im}\, G_{n\mathbf{k}}(\omega). \tag{1}$$

In the cumulant expansion the Green's function is obtained in the time domain, starting from the the interaction picture[1–4]:

$$G_{n\mathbf{k}}(t) = i\, \exp\left[-i\varepsilon_{n\mathbf{k}} t/\hbar + C_{n\mathbf{k}}(t)\right], \tag{2}$$

where $C_{n\mathbf{k}}(t)$ is the cumulant function. By taking the Fourier transform of this expression to the frequency domain and inserting it in Supplementary Eq. (1) we obtain Eq. (1) of the main text. In order to obtain an expression for the cumulant which is amenable to computation, it is customary to expand the exponential in powers of the cumulant: $G_{n\mathbf{k}} = G^0_{n\mathbf{k}}\left[1 + C_{n\mathbf{k}} + C^2_{n\mathbf{k}}/2 + \cdots\right]$, where $G^0_{n\mathbf{k}}(t) = i\, \exp\left(-i\varepsilon_{n\mathbf{k}} t/\hbar\right)$ is the Green's function in absence of electron-phonon interactions. Alternatively, the Green's function can be obtained from the Dyson equation $G_{n\mathbf{k}} = G^0_{n\mathbf{k}} + G^0_{n\mathbf{k}}\varSigma_{n\mathbf{k}}G^0_{n\mathbf{k}} + G^0_{n\mathbf{k}}\varSigma_{n\mathbf{k}}G^0_{n\mathbf{k}}\varSigma_{n\mathbf{k}}G^0_{n\mathbf{k}} + \cdots$. By comparing these expansions term-by-term one finds an explicit expression for the cumulant function, which is given in the Methods and is reproduced here for completeness:

$$C_{n\mathbf{k}}(t) = \frac{1}{\pi\hbar}\int_{-\infty}^{\infty} \mathrm{d}\omega\, \frac{\mathrm{Im}\,\varSigma_{n\mathbf{k}}(\varepsilon_{n\mathbf{k}}/\hbar - \omega)}{(\omega - i\eta)^2}\, e^{(i\omega+\eta)t}\, \theta(\varepsilon_\mathrm{F} - \varepsilon_{n\mathbf{k}} + \hbar\omega). \tag{3}$$

In practical calculations the exact self-energy $\varSigma_{n\mathbf{k}}$ is replaced by the best available approximation, which is the Migdal expression given in Eq. (2) of the main text. A rigorous derivation of the cumulant expansion and a discussion of its advantages and limitations can be found in Supplementary Refs. 4, 5. In particular, in Supplementary Ref. 4 it is shown that the choice of seeding the self-energy calculated with the first non-crossing diagram also guarantees that no overcounting of correlated higher-order contributions is introduced in the theory. The inclusion of crossing diagrams in the evaluation of the Green's function results from the time orderings of the t variables in the cumulant expansion.

The spectral function given in Eq. (1) of the main text yields multiple bosonic satellites, one for each term in the Taylor expansion of $\exp[C_{n\mathbf{k}}(t)]$. A convenient expression for the case of a

single satellite was derived in Supplementary Ref. 3. In this work we extended the method of Supplementary Ref. 3 to the case of multiple satellites. Following Supplementary Ref. 3, we write the spectral function as:

$$A(\mathbf{k},\omega) = \sum\nolimits_n \left[A^{\rm QP}_{n\mathbf{k}}(\omega) + A^{\rm QP}_{n\mathbf{k}}(\omega) * A^{\rm S1}_{n\mathbf{k}}(\omega) + A^{\rm QP}_{n\mathbf{k}}(\omega) * A^{\rm S2}_{n\mathbf{k}}(\omega) + \cdots \right], \tag{4}$$

where $*$ indicates the convolution. In the last expression the quasiparticle contribution $A^{\rm QP}_{n\mathbf{k}}(\omega)$ is defined as:

$$A^{\rm QP}_{n\mathbf{k}}(\omega) = \frac{2}{\pi} \frac{|{\rm Im}\Sigma_{n\mathbf{k}}(\varepsilon_{n\mathbf{k}})|}{[\hbar\omega - \varepsilon_{n\mathbf{k}} - {\rm Re}\Sigma_{n\mathbf{k}}(\varepsilon_{n\mathbf{k}})]^2 + [{\rm Im}\Sigma_{n\mathbf{k}}(\varepsilon_{n\mathbf{k}})]^2}, \tag{5}$$

and the satellite contributions associated with one-phonon and two-phonon processes are:

$$A^{\rm S1}_{n\mathbf{k}}(\omega) = \int_{-\infty}^{\infty} dt\, e^{i\omega t} C_{n\mathbf{k}}(t), \qquad A^{\rm S2}_{n\mathbf{k}}(\omega) = \int_{-\infty}^{\infty} dt\, e^{i\omega t} \frac{1}{2} C_{n\mathbf{k}}(t) C_{n\mathbf{k}}(t)\,. \tag{6}$$

The function $A^{\rm S1}$ can be written in terms of the electron-phonon self-energy by combining Supplementary Eqs. (6) and (3) and carrying out the Fourier transform. This step was performed in Supplementary Ref. 3:

$$A^{\rm S1}_{n\mathbf{k}}(\omega) = \frac{\beta_{n\mathbf{k}}(\omega) - \beta_{n\mathbf{k}}(\varepsilon_{n\mathbf{k}}) - (\omega - \varepsilon_{n\mathbf{k}}/\hbar) \left.\dfrac{\partial \beta_{n\mathbf{k}}}{\partial \omega}\right|_{\varepsilon_{n\mathbf{k}}/\hbar}}{(\hbar\omega - \varepsilon_{n\mathbf{k}})^2}, \tag{7}$$

with

$$\beta_{n\mathbf{k}}(\omega) = \frac{1}{\pi} {\rm Im}\, \Sigma_{n\mathbf{k}}(\varepsilon_{n\mathbf{k}}/\hbar - \omega) \theta(\varepsilon_{\rm F}/\hbar - \omega). \tag{8}$$

In order to obtain the contribution of the second satellite, we note that $A^{\rm S1}_{n\mathbf{k}}(\omega)$ is the Fourier transform of $C_{n\mathbf{k}}(t)$, therefore the expression for $A^{\rm S2}_{n\mathbf{k}}(\omega)$ in Supplementary Eq. (6) can be rewritten using the convolution theorem:

$$A^{\rm S2}_{n\mathbf{k}} = \frac{1}{2} A^{\rm S1}_{n\mathbf{k}} * A^{\rm S1}_{n\mathbf{k}}. \tag{9}$$

By combining Supplementary Eqs. (9) and (4) we obtain the expression used in our calculations:

$$A(\mathbf{k},\omega) = \sum\nolimits_n \left[A^{\rm QP}_{n\mathbf{k}}(\omega) + A^{\rm S1}_{n\mathbf{k}}(\omega) * A^{\rm QP}_{n\mathbf{k}}(\omega) + \frac{1}{2} A^{\rm S1}_{n\mathbf{k}}(\omega) * A^{\rm S1}_{n\mathbf{k}}(\omega) * A^{\rm QP}_{n\mathbf{k}}(\omega) + \cdots \right]. \tag{10}$$

From Supplementary Eq. (10) we can extrapolate the general expression for the case of many satellites:

$$A(\mathbf{k},\omega) = \sum\nolimits_n \left[1 + A^{\rm S1}_{n\mathbf{k}}(\omega) * + \frac{1}{2} A^{\rm S1}_{n\mathbf{k}}(\omega) * A^{\rm S1}_{n\mathbf{k}}(\omega) * + \cdots \right] A^{\rm QP}_{n\mathbf{k}}(\omega) = \sum\nolimits_n e^{A^{\rm S1}_{n\mathbf{k}}(\omega) *} A^{\rm QP}_{n\mathbf{k}}(\omega). \tag{11}$$

When considering a drastically simplified model system consisting of dispersionless electrons and an Einstein phonon spectrum, this last expression reduces to the well-known Lang-Firsov series of polaron satellites[1,6]. In fact, by setting $A^{\mathrm{QP}} = Z\delta(\omega)$ and $A^{\mathrm{S1}} = \lambda\delta(\omega - \omega_{\mathrm{ph}})$, Supplementary Eq. (11) gives $A(\omega) = Z\sum_{m=0}^{\infty}(\lambda^m/m!)\,\delta(\omega - m\,\omega_{\mathrm{ph}})$. By further requiring the normalization of the spectral function, that is $\int A(\omega)\,\mathrm{d}\omega = 1$, we obtain $Z = e^{-\lambda}$ and the Lang-Firsov expression is recovered. In our *ab initio* calculations we truncate Supplementary Eq. (11) to the second order. In practice we first evaluate the quasiparticle and satellite contributions, and then we perform two successive numerical convolutions in order to obtain the two satellites which are seen in the experiments.

Supplementary Note 2 | Mass enhancement and Fröhlich coupling constant

The mass enhancement parameters obtained from the quasiparticle bands along the $\Gamma\Sigma$, ΓX, and ΓZ directions are $\lambda = 0.73$, 0.73, and 0.74 at the lowest doping; $\lambda = 0.70$ in each direction at the intermediate doping; and $\lambda = 0.20$, 0.20, and 0.18 at the highest doping. The mass enhancement can also be calculated directly from the energy derivative of the real part of the electron self-energy at the Fermi level, $\lambda_{\mathbf{k}} = -\partial \mathrm{Re}\Sigma_{\mathbf{k}}(\omega)/\partial\omega|_{\omega=\varepsilon_{\mathrm{F}}}$[7]. We checked that the values thus obtained fall within less than 10% of the ones listed above, the average over the Fermi surface being $\lambda = 0.68$, 0.65 and 0.19 for the three values of doping considered. This approach also validates our calculations of the contribution to λ arising from different phonon modes presented in the analysis of Fig. 2a in the main text.

From the mass enhancement parameter it is also possible to obtain the quasiparticle strength Z as $Z = 1/(1+\lambda)$ [7]. Using the values of λ reported in Fig. 1g-i we obtain $Z = 0.58$, 0.59, and 0.83 with increasing doping. Our calculated quasiparticle strength at intermediate doping overestimates the experimentally-determined value, $Z = 0.36$ [8]. We attribute this difference to extrinsic losses not accounted for in our calculations; these losses are known to transfer spectral weight to the satellites[3,9,10].

In polar materials it is customary to describe the coupling to a dispersionless LO phonon via the

dimensionless Fröhlich coupling constant α:

$$\alpha = \frac{e^2}{\hbar}\left(\frac{m_\mathrm{b}}{2\hbar\omega_\mathrm{LO}}\right)^{1/2}\left(\frac{1}{\epsilon_\infty} - \frac{1}{\epsilon_0}\right), \tag{12}$$

where m_b is the band mass, $\hbar\omega_\mathrm{LO}$ the energy of the LO phonon, ϵ_0 and ϵ_∞ the static and high-frequency permittivities, respectively. We evaluate Supplementary Eq. (12) using the experimental values of ω_LO, ϵ_0, and ϵ_∞ from Supplementary Ref. 11. For the effective masses we use our DFT calculations since no accurate experimental values are available: $m_\perp = 0.40\, m_\mathrm{e}$ and $m_\parallel = 4.03\, m_\mathrm{e}$. By using these parameters in Supplementary Eq. (12) we find the experimental Fröhlich constants $\alpha_\perp = 1.0$ and $\alpha_\parallel = 3.4$, and their isotropic average $\alpha = 1.8$. The Fröhlich constant α can also be obtained from *ab initio* calculations of the electron-phonon matrix elements, following Supplementary Ref. 12. In this case we calculate $\alpha_\perp = 0.9$, $\alpha_\parallel = 3.0$, and the isotropic average $\alpha = 1.6$. These values are in excellent agreement with experiment, therefore our description of Fröhlich coupling in anatase TiO_2 is expected to be highly accurate. We note that the anisotropy of the Fröhlich coupling constant $\alpha_\perp$ and $\alpha_\parallel$ does not stem from anisotropic electron-phonon interactions, but rather from the strong anisotropy in the band masses, which enter α as seen in Supplementary Eq. (12).

For weak and intermediate couplings, the polaron effective mass is commonly estimated using $m^* = m_\mathrm{b}(1 - \alpha/6)^{-1}$ [13,14]. While this procedure is adequate for isotropic crystals, it cannot be used in the present case of TiO_2. In fact, if we use the isotropic average of the coupling constant, then we underestimate m^* with respect to experiment[8]. On the other hand, if we consider $\alpha_\perp$ and $\alpha_\parallel$ separately, then we obtain a strongly anisotropic mass enhancement, which is not consistent with our many-body calculations. These observations indicate that, in the case of anisotropic crystals, the Fröhlich constant α should be used with caution.

Supplementary Note 3 | Dielectric screening

In this work we calculate the additional screening arising from the charge carriers using the random phase approximation (RPA) for the homogeneous electron gas, that is the Lindhard screening. This choice is motivated by the fact that we must evaluate the screening for millions of electron-phonon matrix elements, and explicit *ab initio* calculations of the RPA screening for such a dense Brillouin

zone grid are not currently feasible. The use of the Lindhard model is justified by the fact that the system under consideration lies in the high-density electron-gas limit. To confirm this point we evaluate the Wigner-Seitz radius r_s given by $r_s = (4\pi n a_0^{*3}/3)^{-1/3}$, where n is the doping density and $a_0^* = \hbar^2\epsilon_0/(e^2 m_{\rm b})$[15]. Using the band effective mass $m_{\rm b}$ and the static dielectric constant ϵ_0 of anatase we obtain $r_s = 1.6$ for the lowest doping level 5×10^{18} cm^{-3}. This value is comparable or even smaller than what found in simple metals, therefore the use of the electron gas model to describe doped carriers is justified. Moreover, the electronic bands are to a good approximation parabolic in the energy range considered in this work; this is seen by comparing the *ab initio* density of states with the parabolic band model, Supplementary Fig. 2c. Therefore the use of a Lindhard function is justified, and we expect this choice to be very accurate in the present case.

Supplementary Note 4 | Polaron wavefunction

In order to calculate the wavefunction of a polaron, we follow the approach of Supplementary Ref. 15 and express the many-body electron-phonon state using Rayleigh-Schrödinger perturbation theory. We consider a system at zero temperature and with a single electron added to the bottom of the conduction band. The resulting expression is:

$$\tilde{\psi}_{n\mathbf{k}}(\mathbf{r};\{\boldsymbol{\tau}_\kappa\}) = \psi_{n\mathbf{k}}(\mathbf{r})\,|0_{\rm p}\rangle + \frac{1}{\sqrt{N_\mathbf{q}}}\sum_{m\nu\mathbf{q}} \frac{g_{mn\nu}(\mathbf{k},\mathbf{q})\psi_{m\mathbf{k}+\mathbf{q}}(\mathbf{r})}{\varepsilon_{n\mathbf{k}} - \varepsilon_{m\mathbf{k}+\mathbf{q}} - \omega_{\mathbf{q}\nu}}\,\hat{a}^\dagger_{-\mathbf{q}\nu}\,|0_{\rm p}\rangle\,, \tag{13}$$

where $\{\boldsymbol{\tau}_\kappa\}$ are the nuclear coordinates, $\hat{a}^\dagger_{-\mathbf{q}\nu}$ is a phonon creation operator, and $|0_{\rm p}\rangle$ is the ground state with no phonons. The Brillouin zone is discretized using a uniform grid with $N_\mathbf{q}$ phonon wavevectors. Since the atomic displacements are smaller than characteristic interatomic distances, we can simplify the above expression by replacing $\hat{a}^\dagger_{-\mathbf{q}\nu}$ (which is a function of the normal mode coordinates) by its average over a given electron-phonon state[15]. In order to determine a lower bound to the polaron radius, we evaluate this expectation value by considering an electronic wavefunction localized at the center of the reference frame, $\mathbf{r} = 0$. The result is:

$$\langle \hat{a}^\dagger_{-\mathbf{q}\nu}\rangle = \frac{1}{\sqrt{N_\mathbf{q}}}\sum_{m'} \frac{g^*_{m'n\nu}(\mathbf{k},\mathbf{q})}{\varepsilon_{n\mathbf{k}} - \varepsilon_{m'\mathbf{k}+\mathbf{q}} - \omega_{\mathbf{q}\nu}}. \tag{14}$$

By replacing $\langle \hat{a}^{\dagger}_{-\mathbf{q}\nu} \rangle$ inside Supplementary Eq. (13) we have:

$$\tilde{\psi}_{n\mathbf{k}}(\mathbf{r};\{\boldsymbol{\tau}_\kappa\}) = \frac{e^{i\mathbf{k}\cdot\mathbf{r}}}{\sqrt{\Omega}} \left[u_{n\mathbf{k}}(\mathbf{r}) + \frac{1}{N_\mathbf{q}} \sum_{m\nu\mathbf{q}} \frac{g_{mn\nu}(\mathbf{k},\mathbf{q}) u_{m\mathbf{k}+\mathbf{q}}(\mathbf{r}) e^{i\mathbf{q}\cdot\mathbf{r}}}{\varepsilon_{n\mathbf{k}} - \varepsilon_{m\mathbf{k}+\mathbf{q}} - \omega_{\mathbf{q}\nu}} \sum_{m'} \frac{g^*_{m'n\nu}(\mathbf{k},\mathbf{q})}{\varepsilon_{n\mathbf{k}} - \varepsilon_{m'\mathbf{k}+\mathbf{q}} - \omega_{\mathbf{q}\nu}} \right] |0_\mathrm{p}\rangle \,. \tag{15}$$

The expression for $\tilde{\psi}_{n\mathbf{k}}$ given in the Methods was obtained from Supplementary Eq. (15) by considering the normalization of the wavefunction and by retaining the long-wavelength part of the Fröhlich vertex, so that $g_{mn\nu}(\mathbf{k},\mathbf{q}) \rightarrow g_{mn\nu}(\mathbf{k},\mathbf{q})\,\delta_{mn}$. We already demonstrated that this approximation is very accurate for anatase TiO_2[12]. Since the main contribution to Supplementary Eq. (13) arises from long-wavelength optical phonons, we also replace $u_{m\mathbf{k}+\mathbf{q}}(\mathbf{r})$ by $u_{m\mathbf{k}}(\mathbf{r})$, in the spirit of the $\mathbf{k}\cdot\mathbf{p}$ approximation.

Our choice of calculating the polaron wavefunction within Rayleigh-Schrödinger perturbation theory is justified since this theory is valid in the range $\alpha \leq 5$ (see Supplementary Ref. 15, p. 516), and the largest Fröhlich coupling constant in anatase TiO_2 is $\alpha_{\|} = 3.0$ (see Supplementary Note 2).

We note that the present many-body approach for calculating the polaron wavefunction differs significantly from DFT calculations of excess electrons in oxides using large supercells[16–19]. In fact in the present case we employ a quantum description of both electrons and nuclei, and a dynamical (non-adiabatic) description of their interactions. In contrast, supercell DFT calculations describe the nuclei as classical particles and decouple electronic and nuclear degrees of freedom using the Born-Oppenheimer adiabatic approximation. These approximations are not justified in the case of TiO_2, and have led to results which are very sensitive to the DFT exchange and correlation functional and the size of the supercell. Our present perturbative approach does not suffer from these shortcomings.

Supplementary References

1. Langreth, D. C. Singularities in the X-ray spectra of metals. *Phys. Rev. B* **1**, 471–477 (1970).
2. Hedin, L. Effects of recoil on shake-up spectra in metals. *Phys. Scr.* **21**, 477–480 (1980).
3. Aryasetiawan, F., Hedin, L. & Karlsson, K. Multiple plasmon satellites in Na and Al spectral functions from *ab initio* cumulant expansion. *Phys. Rev. Lett.* **77**, 2268–2271 (1996).
4. Gumhalter, B., Kovač, V., Caruso, F., Lambert, H. & Giustino, F. On the combined use of GW approximation and cumulant expansion in the calculations of quasiparticle spectra: The paradigm of Si valence bands. *Phys. Rev. B* **94**, 035103 (2016).
5. Zhou, J. S. *et al.* Dynamical effects in electron spectroscopy. *J. Chem. Phys.* **143**, 184109 (2015).
6. Berciu, M. Green's function of a dressed particle. *Phys. Rev. Lett.* **97**, 036402 (2006).
7. Grimvall, G. *The Electron-Phonon Interaction in Metals* (North-Holland, New York, 1981).
8. Moser, S. *et al.* Tunable polaronic conduction in anatase TiO_2. *Phys. Rev. Lett.* **110**, 196403 (2013).
9. Bardyszewski, W. & Hedin, L. A new approach to the theory of photoemission from solids. *Phys. Scr.* **32**, 439–450 (1985).
10. Kas, J. J., Rehr, J. J. & Curtis, J. B. Particle-hole cumulant approach for inelastic losses in x-ray spectra. *Phys. Rev. B* **94**, 035156 (2016).
11. Gonzalez, R. J., Zallen, R. & Berger, H. Infrared reflectivity and lattice fundamentals in anatase TiO_2. *Phys. Rev. B* **55**, 7014–7017 (1997).
12. Verdi, C. & Giustino, F. Fröhlich electron-phonon vertex from first principles. *Phys. Rev. Lett.* **115**, 176401 (2015).
13. Fröhlich, H. Electrons in lattice fields. *Adv. Phys.* **3**, 325–361 (1954).
14. Mishchenko, A. S., Prokof'ev, N. V., Sakamoto, A. & Svistunov, B. V. Diagrammatic quantum Monte Carlo study of the Fröhlich polaron. *Phys. Rev. B* **62**, 6317–6336 (2000).

15. Mahan, G. D. *Many-Particle Physics* (Plenum, New York, 1981).

16. Di Valentin, C. & Selloni, A. Bulk and surface polarons in photoexcited anatase TiO_2. *J. Phys. Chem. Lett.* **2**, 2223–2228 (2011).

17. Deák, P., Aradi, B. & Frauenheim, T. Quantitative theory of the oxygen vacancy and carrier self-trapping in bulk TiO_2. *Phys. Rev. B* **86**, 195206 (2012).

18. Setvin, M. *et al.* Direct view at excess electrons in TiO_2 rutile and anatase. *Phys. Rev. Lett.* **113**, 086402 (2014).

19. Spreafico, C. & VandeVondele, J. The nature of excess electrons in anatase and rutile from hybrid DFT and RPA. *Phys. Chem. Chem. Phys.* **16**, 26144–26152 (2014).
